# Identification of novel post-transcriptional features in olfactory receptor family mRNAs

**DOI:** 10.1093/nar/gkv324

**Published:** 2015-04-23

**Authors:** Eleen Y. Shum, Josh L. Espinoza, Madhuvanthi Ramaiah, Miles F. Wilkinson

**Affiliations:** 1 Department of Reproductive Medicine, School of Medicine, University of California, San Diego, La Jolla, CA 92093-0695, USA; 2 Institute of Genomic Medicine, University of California, San Diego, La Jolla, CA 92093, USA

## Abstract

Olfactory receptor (*Olfr*) genes comprise the largest gene family in mice. Despite their importance in olfaction, how most *Olfr* mRNAs are regulated remains unexplored. Using RNA-seq analysis coupled with analysis of pre-existing databases, we found that *Olfr* mRNAs have several atypical features suggesting that post-transcriptional regulation impacts their expression. First, *Olfr* mRNAs, as a group, have dramatically higher average AU-content and lower predicted secondary structure than do control mRNAs. Second, *Olfr* mRNAs have a higher density of AU-rich elements (AREs) in their 3′UTR and upstream open reading frames (uORFs) in their 5 UTR than do control mRNAs. Third, *Olfr* mRNAs have shorter 3′ UTR regions and with fewer predicted miRNA-binding sites. All of these novel properties correlated with higher *Olfr* expression. We also identified striking differences in the post-transcriptional features of the mRNAs from the two major classes of *Olfr* genes, a finding consistent with their independent evolutionary origin. Together, our results suggest that the *Olfr* gene family has encountered unusual selective forces in neural cells that have driven them to acquire unique post-transcriptional regulatory features. In support of this possibility, we found that while *Olfr* mRNAs are degraded by a deadenylation-dependent mechanism, they are largely protected from this decay in neural lineage cells.

## INTRODUCTION

Olfactory receptors (OLFRs) are G protein-coupled receptors (GPCRs) essential for odor detection in olfactory sensory neurons (OSNs). These receptors are encoded by the largest gene family in mice, occupying ∼2% of the protein-coding genome (1–3). *Olfr* genes are divided into 2 classes, each of which has a different evolutionary origin: class-I receptors are thought to be derived from ancestral fish and evidence suggests that class-II receptors evolved from ancestral amphibians (3). These two *Olfr* gene classes are responsible for generating receptors that detect different odorants; e.g. it has been shown that class-I OLFRs preferentially detect predator-related odorants (4).

*Olfr* genes are regulated in a unique manner. Only a single *Olfr* gene allele from ∼1000 *Olfr* gene choices is selected to be expressed in a given OSN (5–8). The *Olfr* gene selected by each OSN is not only responsible for detecting odorants in the olfactory epithelium (OE), but it also directs the axons of OSNs that express the same *Olfr* gene to converge into the same glomerulus in the olfactory bulb (9,10). By controlling both axon guidance and receptor expression, this ‘1-receptor, 1-cell' rule provides the foundation by which the olfactory system distinguishes different odorants (1,3,11). How precisely this 1-receptor, 1-cell rule is implemented at the molecular level remains enigmatic. In principal, it appears to be largely dictated by a selective transcriptional mechanism in which one *Olfr* gene is transcriptionally activated and all other *Olfr* genes are transcriptionally repressed in a given OSN. Likely to be involved are transcription factors that regulate *Olfr* gene expression, including the LHX2 LIM/homeobox transcription factor and members of the OLF-1/EBF (O/E) helix-loop-helix (HLH) family (12–14). The regulation of *Olfr* gene choice may also be dictated by epigenetic signatures that correlate with transcriptional activity (15,16).

While considerable progress has been defining transcriptional mechanisms acting on *Olfr* genes, little is known about post-transcriptional mechanisms regulating *Olfr* mRNAs. This is a large gap in the field given that post-transcriptional regulation has the potential to be critical for regulation of OLFR expression. For example, selective RNA decay mechanisms could contribute to the ‘1-receptor, 1-cell rule’ by degrading non-selected *Olfr* mRNAs that are expressed from incompletely silenced *Olfr* genes. Post-transcriptional mechanisms also have the potential to control *Olfr* mRNA levels during OSN development, as well as in response to acute exposure to odorants.

Post-transcriptional regulation is typically directed toward the 5′ and 3′ untranslated regions (UTRs) of mRNAs, as they house a plethora of *cis* elements that impact mRNA stability and translation. For example, UTRs harbor sequence motifs and secondary structures that recruit ribosomes and RNA-binding proteins (RBPs) to govern rates of mRNA decay and translation (17–19). Also recruited to UTRs, particularly to 3′ UTRs, are microRNAs (miRNAs), which are short RNAs that elicit translational repression, mRNA destabilization, or both (20). None of these features have been investigated in *Olfr* mRNAs.

To address post-transcriptional regulatory mechanisms that regulate *Olfr* transcripts, it is critical to first define *Olfr* mRNA sequences. Zhang *et al*. and Shiao *et al*. detected the expression of ∼800–1200 *Olfr* mRNAs in the OE using a custom microarray and RNA-seq, respectively, but they did not define the 5′ and 3′ termini of these transcripts or identifying alternative isoforms (21,22). Other studies have used transcription start-site mapping to identify the 5′ UTR and promoter regions of ∼200 *Olfr* mRNAs (23–26) and one study screened cDNA libraries to identify promoter and 5′ UTR sequences of ∼400 *Olfr* mRNAs (27). In our study, we employed RNA-seq analysis to analyze *Olfr* mRNAs with respect to their post-transcriptional features. Our analysis revealed that *Olfr* mRNAs tend to have several unique features, including a short 3′ UTR, high AU-content, and a high density of AREs and uORFs. After our manuscript describing this work was submitted, another paper was published that characterized *Olfr* mRNAs using RNA-seq analysis (28). While this Ibarra-Soria *et al*. paper did not focus on post-transcriptional features, it provided us an opportunity to examine such features using another *Olfr* data set. As described herein, the data sets from this paper corroborated what we determined with our *Olfr* data sets. In summary, we have uncovered unusual post-transcriptional features that are unique to the *Olfr* gene family, thereby shining light into how these genes are potentially regulated in OSNs. In support, we provide *in vitro* evidence that *Olfr* mRNAs are post-transcriptionally regulated in a neuronal-specific manner.

## MATERIALS AND METHODS

### Compiling the *Olfr* mRNA transcriptome

RNA-seq analysis was performed on two 8 week-old *C57BL/6* female OE littermates, who were housed in accordance with UCSD IACUC policies. In brief, dissected OE RNA was isolated using TriZOL (Life Technologies), followed by a secondary purification step using RNeasy columns (Qiagen). RNA-seq was performed at the UCSD Biogem Core. Total RNA was assessed for quality using an Agilent Bioanalyzer, and samples determined to have an RNA Integrity Number (RIN) of at least 8 or greater were used to generate RNA libraries using Illumina's TruSeq RNA Sample Prep Kit following manufacturer specifications, with the RNA fragmentation time adjusted to 5 min. 1 ug of total RNA was used per sample. RNA libraries were multiplexed and sequenced at 10 pM with 50 basepair (bp) single end reads to a depth of approximately 40 million reads per sample on an Illumina HiSeq2000. The OE transcriptome was extracted using the Tuxedo suite, encompassing Bowtie2, Tophat2 and Cufflinks programs (29,30) using the ‘novel gene discovery mode’ as outlined in the Tuxedo suite program manuals (30). Specifically using the CuffCompare program, we extracted information such as the nearest Ensembl reference ID, exon start/end positions, chromosome, gene direction and gene symbol from each gene entry. Next, we fetched the CDS of all entries using the nearest reference IDs from the UCSC Table Browser from the NCBIm37/mm9 genome. Using the exon start/end positions for each reference ID entry, we extracted the exon sequences from the mm9 genome, and ligated them together *in silico* for each transcript. For each entry, the entire transcript sequence is subtracted from the known CDS sequence obtained above to identify the 5′ and 3′ UTRs. If a 30-nucleotide sequence was matched for the 5′ and 3′ peripheral regions of the nearest reference coding sequence with the corresponding *in silico* spliced transcript then the transcript was considered complete. For the complete transcripts, the 5′ and 3′ regions flanking the coding sequence were considered 5′ and 3′ UTRs, respectively. Intron sequences were extracted by using the regions between the exon junctions for each transcript. The second set of *Olfr* transcripts were provided by the supplemental data sets provided by Ibarra-Soria *et al*. (28). Only transcripts with both a detectable 5′ UTR and 3′UTR were considered in subsequent analysis.

### GC-content, ARE and miRNA site search

Once extracted, the lengths and GC-content were calculated for 5′/3′ UTRs, coding sequences and introns. To calculate global GC-content for Olfr, control and GPCR groups we created sets for every position, ranging from −50 to +50, flanking the splice-site of each intron. The GC-content was calculated for each of these positions to construct a general global GC analysis for each group of transcripts. To construct sequence logos, we used all sequences flanking splice-sites, ranging from −15 to +15, in UCB's WebLogo application (http://weblogo.berkeley.edu/) (31). The 3′UTR sequences generated during the annotation were used to scan for AREs. We scanned the 3′UTR sequences for the following n-mers identified by AREsite (http://rna.tbi.univie.ac.at/cgi-bin/AREsite.cgi) (32): AUUUA and WWAUUUAWW. W indicates either an adenine or uracil nucleotide. miRNA sites were discovered using TargetScan 6.0 (33–36). In brief, 9 miRNA mature and whole sequences were scanned along all genes from the three gene groups to find putative miRNA binding sites.

### Secondary structure minimum free energy

To select the minimum free energy of the 5′UTR sequences, we used University of Vienna's RNAfold Web Server (http://rna.tbi.univie.ac.at/cgi-bin/RNAfold.cgi) (37) to calculate the folding energy. The 5′UTR sequences used were generated from the RNA-seq reads, Ensembl reference IDs, and the annotation algorithms described above.

### 3′UTR cloning, cell culture and luciferase assay

The 3′ UTRs of *Olfr212, Olfr1226* and *Olfr1242* were amplified by PCR from adult OE cDNA and cloned downstream of the Firefly luciferase ORF (at the SpeI and HindIII restriction enzyme sites) in the pMIR-REPORT vector (Ambion). These reporter constructs were transfected by Lipofectamine 2000 reagent (Life Technologies) into P19 and Neuro2A cells. The P19 cells were cultured in 10% fetal calf serum (Stem Cell Technologies) and MEMα media (Invitrogen); Neuro2A cells were cultured in 10% fetal calf serum (Stem Cell Technologies) and DMEM media (Invitrogen). Also transfected into these cells was the CCR4B expression vector (kindly provided by Jens Lykke-Andersen [UCSD]) and the *Ccr4b* siRNA and the ONTARGETplus siRNA control (ThermoScientific). The Firefly Renilla luciferase reporter was co-transfected as an internal control. Luciferase activity was assayed as previously described (38) using the Dual Luciferase Assay System (Promega). Cellular RNA was harvested using TriZOL (Invitrogen), followed by iScript Reverse Transcription Kit and SYBR Green qPCR Reagent (BioRad), all according to manufacturer's protocol. The level of *Ccr4b* mRNA was measured using the following qPCR primers: UCSD8023: CGCGGAGGAGAATGAGACTA and UCSD8024: GTGCAGTGCTGTCAAGTGTG. The endogenous RNA control *Rpl19* was used and was amplified using the following qPCR primers: UCSD4667: CTGAAGGTCAAAGGGAATGTG and UCSD4668: GGACAGAGTCTTGATGATCTC.

### *In situ* hybridization

*In situ* hybridization was performed as previously described previously (38) using 10 μm fresh frozen OE cryosections and LNA probes against *miR-741, miR-9* and *miR-128* (Exigen).

### Identifying uORFs

The 5′ UTR sequences identified through our annotation process (described above when compiling *Olfr* transcript above) were used to scan for upstream open reading frames that contained particular nucleotides from the Kozak consensus sequence critical for initiating translation (39). In particular, we only considered uORFs ≥ 30 nt long that had a purine at the −3 position or a guanine at the +1 position (relative to the A in the AUG initiation codon [+1]). To reduce the probability of identifying uORFs that can re-initiate translation (and thus escape NMD), one additional criteria is the uORF must not contain the main open reading frame.

### Programming language used

Algorithms were generated with the Python programming language (version 2.7.6). Data analysis was performed using both R and GraphPad PRISM.

## RESULTS AND DISCUSSION

### Genome-wide identification of class I and II *Olfr* transcripts

**Figure 1.**
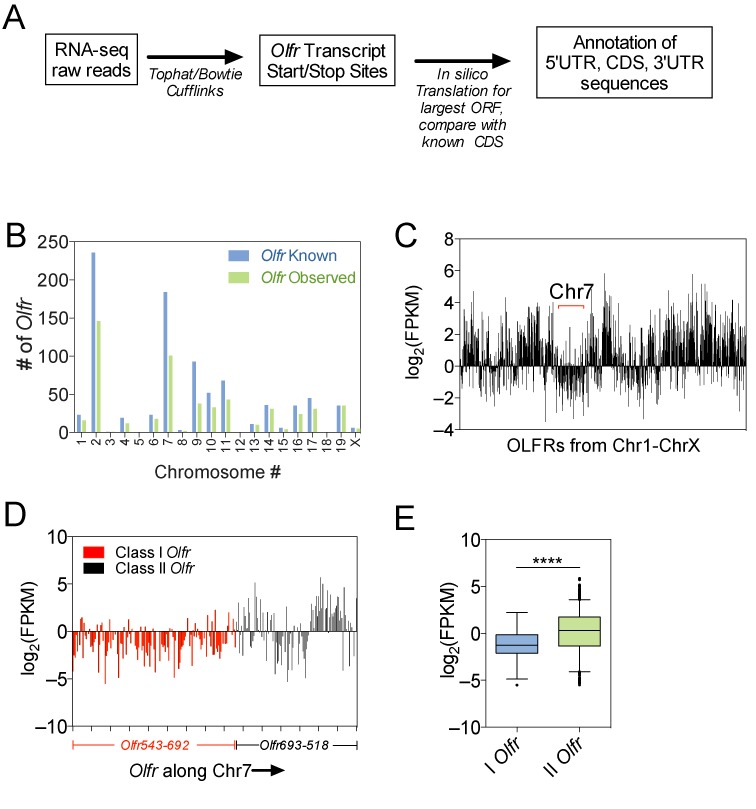
RNA-seq analysis of *Olfr* mRNAs in the mouse adult OE. (**A**) Schematic of workflow used to assemble the *Olfr* transcriptome. (**B**) Full-length *Olfr* mRNA transcripts detected by our RNA-seq analysis versus all known *Olfr* genes in each mouse chromosome. (**C**) Bar graph of the expression level of individual *Olfr* genes plotted sequentially along all mouse chromosomes, expressed as Fragments Per Kilobase of transcript per Million mapped reads (FPKM). (**D**) Bar graph displaying the expression level of individual *Olfr* genes along chromosome 7. (**E**) Boxplot of average expression level of class-I versus -II *Olfr* transcripts. **** *P* < 0.0001.

*Olfr* transcripts were defined using RNA-seq data from adult mice OE as outlined in Figure 1A. We detected reads from 1101 *Olfr* genes, but there were insufficient reads from some of these *Olfr* genes to accurately determine their 5′ and 3′ termini (data not shown). To exclusively annotate *Olfr* mRNAs that encode functional proteins, we only analyzed transcripts with RNA-seq reads matching the coding sequence (CDS) of known OLFR proteins, as defined in the Ensembl database. Using this criterion, we identified 805 full-length mRNAs encoded by 554 *Olfr* genes (Supplementary Table S1). The alternative isoforms included those with different 5′ and 3′ UTR regions and those generated by alternative splicing (Supplementary Table S2). The *Olfr* transcripts we identified emanate from *Olfr* genes distributed on the various chromosomes known to have *Olfr* gene clusters (Figure 1B; 27), suggesting that our analysis was not biased for a particular sector of the genome. Analysis of the reads corresponding to individual *Olfr* genes showed they exhibited considerable heterogeneity in expression (Figure 1C and D), confirming findings from other studies on *Olfr* mRNAs (27,28).

We observed that class-I *Olfr* genes tend to be expressed at strikingly lower level than most class-II *Olfr* genes (Figure 1C–E). A likely explanation is that class-I *Olfr* genes are selected to be expressed less often than are class-II *Olfr* genes in individual OSNs, as class-I *Olfr* genes comprise only ∼10% of all *Olfr* genes (40). In further support of this possibility, class-I OLFR-expressing OSNs are only found in the dorsal zone of the OE, whereas class-II OLFR-expressing OSNs are present in virtually all regions of the OE (41,42). A non-mutually exclusive explanation for the lower levels of class-I *Olfr* mRNAs in the OE is that class-I *Olfr* genes are transcribed at a lower rate *per OSN* than are class-II *Olfr* genes. Since class-I *Olfr* genes have a distinct evolutionary origin to that of class-II *Olfr* genes (3), they may have distinct promoters that support lower levels of transcription than do class-II promoters. Finally, it is also possible that class-I *Olfr* mRNAs are intrinsically less stable than are class-II *Olfr* mRNAs.

### *Olfr* mRNAs tend to have short 3′ UTRs

**Figure 2.**
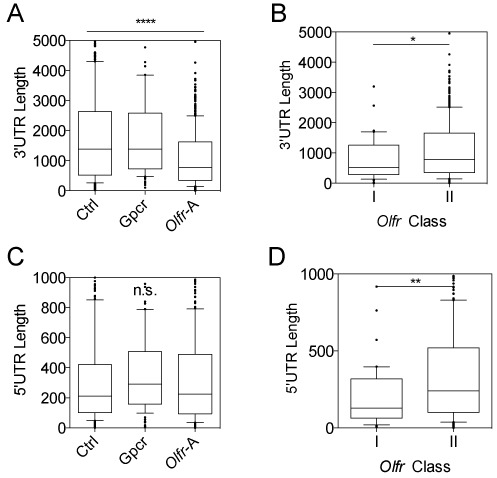
Most *Olfr* transcripts have short 3′ UTRs. (**A**, **B**) Boxplots of 3′ UTR length. (**C**, **D)** Boxplots of 5′ UTR length. *P < 0.05, **P < 0.01, ****P < 0.0001; n.s. not statistically significant.

To understand how *Olfr* mRNA characteristics potentially impact their expression, we analyzed *Olfr* mRNAs we defined by RNA-seq (Figure 2, Supplementary Figure S1A–B, Supplementary Table S3), as *Olfr* trancripts on publically available databases such as Ensembl and RefSeq have inaccuracies. Unless otherwise noted, all data analysis below was performed in *Olfr* mRNAs we identified through our RNA-seq analysis. As a basis of comparison, we examined two other gene groups: (i) *Gpcr* genes encoding 92 non-OLFR GPCR proteins defined by the International Union of Basic and Clinical Pharmacology Database [IUPHAR-DB] (43) that we found were significantly expressed in the OE, based on our RNA-seq analysis, and (ii) *Ctrl* genes, a randomly selected group of 587 non-*Gpcr* genes expressed in the OE.

This analysis revealed unique features of *Olfr* mRNAs as a group. One unusual feature of *Olfr* mRNAs is 3′ UTR length. The average 3′ UTR length of *Olfr* mRNAs in our data set (‘Olfr A’, 1094 nt) is dramatically shorter than that of the *Ctrl* (1895 nt) and *Gpcr* (1800 nt) group mRNAs (Figure 2A; *P* < 0.0001). *Olfr* mRNAs defined by Ibarra-Soria *et al*. (28) (‘Olfr-B’) also had a shorter 3′ UTR length (1439 nt; *P* < 0.0001) (Supplementary Figure S1C). Both class-I and -II *Olfr* mRNAs had shorter 3′ UTRs than control gene groups, but was most evident for the former (786 nt and 1121 nt average 3′ UTR length, respectively; *P* < 0.05; Figure 2B and Supplementary Table S3). The longer average *Olfr* mRNA length determined by Ibarra-Soria *et al*. compared to that found in our study could be due to variability in sampling conditions or transcriptome assembly algorithm differences, or simply because different mice have different repertoires of *Olfr* mRNAs (22). For example, Ibarra-Soria *et al*. used both males and female mice for their analysis, while our analysis used only female mice (28). Given that males and females have a vastly different profile of *Olfr* expression, this experimental difference could explain some transcriptome annotation differences.

The finding that *Olfr* 3′ UTRs tend to be shorter than control 3′ UTRs (Figure 2A, Supplementary Figure S1C) raised the possibility that there has been strong selection pressure on *Olfr* genes to evade post-transcriptional regulation pathways that target the 3′ UTR. For example, previous work has suggested that polyadenylation site selection shifts during development and cancer progression to differentially expose recognition sites for RNA-binding proteins (RBPs) or miRNAs for regulatory purposes (44–49). Both RBPs and miRNAs dictate translation efficiency and RNA decay rates, and thus mechanisms that control their ability to be recruited to an mRNA are critical for the fate of an mRNA (50). Below, we consider how the unique characteristics of *Olfr* 3′ UTRs, including their short length, might shape the post-transcriptional fate of *Olfr* transcripts.

We also found that there is considerably heterogeneity in the length in *Olfr* mRNAs encoded by different *Olfr* genes (Supplementary Figure S1D). This is largely attributable to the heterogeneity of 3′ UTR length, since the coding region (CDS) length of *Olfr* mRNAs are nearly invariant (Supplementary Figure S1E) and the 5′ UTR region does not contribute much to the variation in *Olfr* mRNAs (Supplementary Figure S1F). Another contributing factor to *Olfr* mRNA length heterogeneity is the difference in the average length of class-I and-II *Olfr* mRNAs; we found that class-I mRNAs are modestly, but significantly shorter than class-II mRNAs (Supplementary Figure S1G).

In contrast to the 3′ UTR, the 5′ UTR was not significantly different in median length in *Olfr* mRNAs (223 nt in *Olfr*-A, 241 nt in *Olfr*-B) as compared to either *Ctrl* (212 nt) or *Gpcr* (242 nt) mRNAs (Figure 2C). However, we found that *Olfr* 5′ UTRs were quite heterogeneous in length, in large part because class-I *Olfr* 5′ UTRs (129 nt) are, on average, half the length of Class-II *Olfr* 5′ UTRs (240 nt, Figure 2D; *P* < 0.01, Supplementary Table S3). Thus, Class-I *Olfr* 5′ UTRs tend to be much shorter than the average length of 5′ UTRs in mRNAs in general. We suggest that this likely reflects the different evolutionary origins and/or unique selection pressures exerted on class-I *Olfr* genes (3). 5′ UTR regions are known to be major sites for regulation of translation efficiency and RNA decay rate (19) and thus class-I *Olfrs* may have been selected to have short 5′ UTR regions over evolutionary time for regulatory purposes.

### *Olfr* mRNAs have few predicted binding sites for OE-expressed miRNAs

We first considered the possibility that *Olfr* mRNAs have short 3′ UTRs to avoid repression by miRNAs. miRNAs are short (18-22 nucleotides) RNAs that bind by complementarity with their mRNA targets and recruit RBPs that lead to mRNA stabilization and/or translational repression (51). miRNAs typically bind to the 3′ UTR region of their target mRNAs and thus there has been considerable interest in identifying mechanisms that dictate which 3′ UTR regions are accessible to miRNA-mediated regulation (44,47,52). In the case of *Olfr* mRNAs, we hypothesized that they were selected to harbor short 3′ UTRs to escape repression mediated by miRNAs.

**Figure 3.**
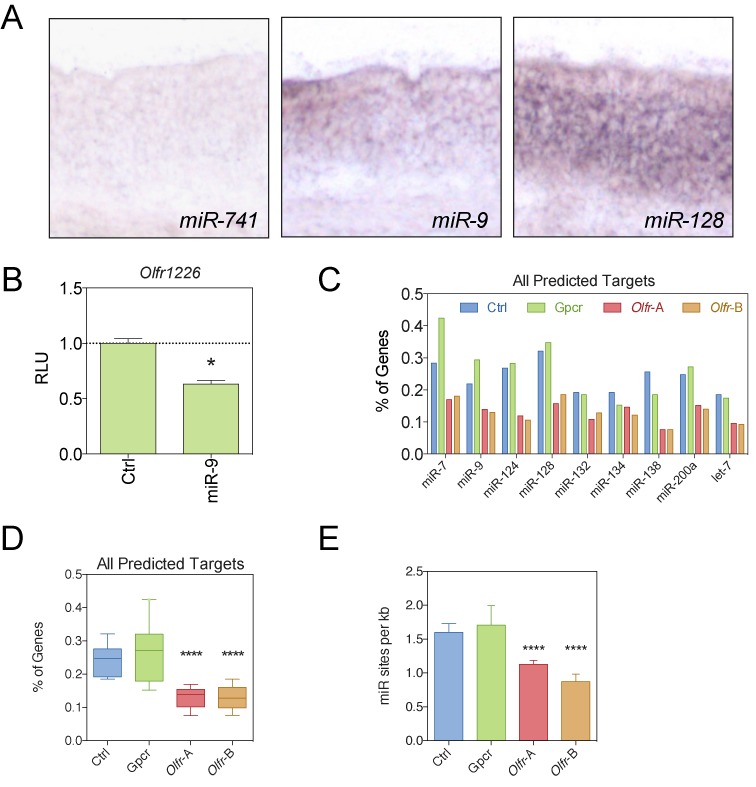
*Olfr* mRNAs tend to have few predicted target sites for neurally expressed miRNAs. (**A**) *In situ* hybridization of neurally expressed miRNAs in the OE. (**B**) Dual luciferase analysis of the effect of the *Olfr1226* 3′ UTR in response to ectopic expression of *miR-9* in Neuro2A cells. (**C**) The percentage of mRNAs from the indicated gene groups with predicted miRNA-binding site defined using the TargetScan algorithm. (**D**) Boxplot representation of the average percentage of miRNA-binding sites determined across all 9 miRNAs, determined from the data in panel **C**. (**E**) Average frequency of miRNA sites per kb of 3′UTR in each gene group. *P < 0.05; ****P < 0.0001.

To test this possibility, we used the miRNA target-prediction program, TargetScan, to screen for miRNA-binding seed sequences in the *Olfr* mRNAs we defined as well as the data set from Ibarra-Soria *et al*. In particular, we used this *in silico* screen to examine the following miRNAs: *miR-200a/miR-141a*, the only miRNA known to have a role in olfactory neurogenesis (53), and eight well-studied neurally expressed miRNAs: *miR-7, -9, -124, -128, -132, -134, -138* and *let-7* (38,54–61). We found that at least 2 of these miRNAs—*miR-9* and *-128*—are expressed in the OSN layer of the OE (Figure 3A). We functionally tested one of the *Olfr* 3′ UTRs (*Olfr1226*) with a predicted miR-9 target site. In support of the accuracy of the TargetScan algorithm, we found that the *Olfr1226* 3′ UTR conferred repression in response to exogenously expressed miR-9 (Figure 3B).

Analysis of predicted target sites revealed less predicted binding sites for 8 out of the 9 miRNAs in *Olfr* transcripts compared to the *Gpcr* and *Ctrl* transcript groups (Figure 3C–D, Supplementary Table S4). As another means to analyze miRNA-regulatory potential, we examined the percentage of mRNAs in a given group predicted to be targeted by the nine neurally expressed miRNAs. We found that a significantly lower percentage of *Olfr* mRNAs had predicted target sites for these nine miRNAs than did the other mRNA groups (*P* < 0.0001; Figure 3C).

We considered the possibility that the only reason that *Olfr* mRNAs have fewer miRNA-target sites than control mRNAs is because their 3′ UTRs are shorter. If this was the case, then the density of miRNA-target sites should be similar between the three groups. Instead, we found that *Olfr* mRNAs had significantly lower density of predicted binding sites for the nine miRNAs than the control groups (Figure 3E). Thus, the low number of predicted miRNA-target sites in *Olfr* mRNAs is not only because they tend to have short 3′ UTRs. Together, these data are consistent with a model in which *Olfr* genes evolved to reduce miRNA-mediated repression by both shortening their 3′ UTRs and reducing the number of miRNA-target sites by mutation. During the course of this analysis, we noted that a relatively large percentage of *Gpcr* genes (>15%) contain predicted *miR-7* and *-128* binding sites (Figure 3C), suggesting that *miR-7, -200a, -9* and *-128*, in particular, may be broad regulators of *Gpcr* mRNAs.

### *Olfr* mRNAs are AU-rich

**Figure 4.**
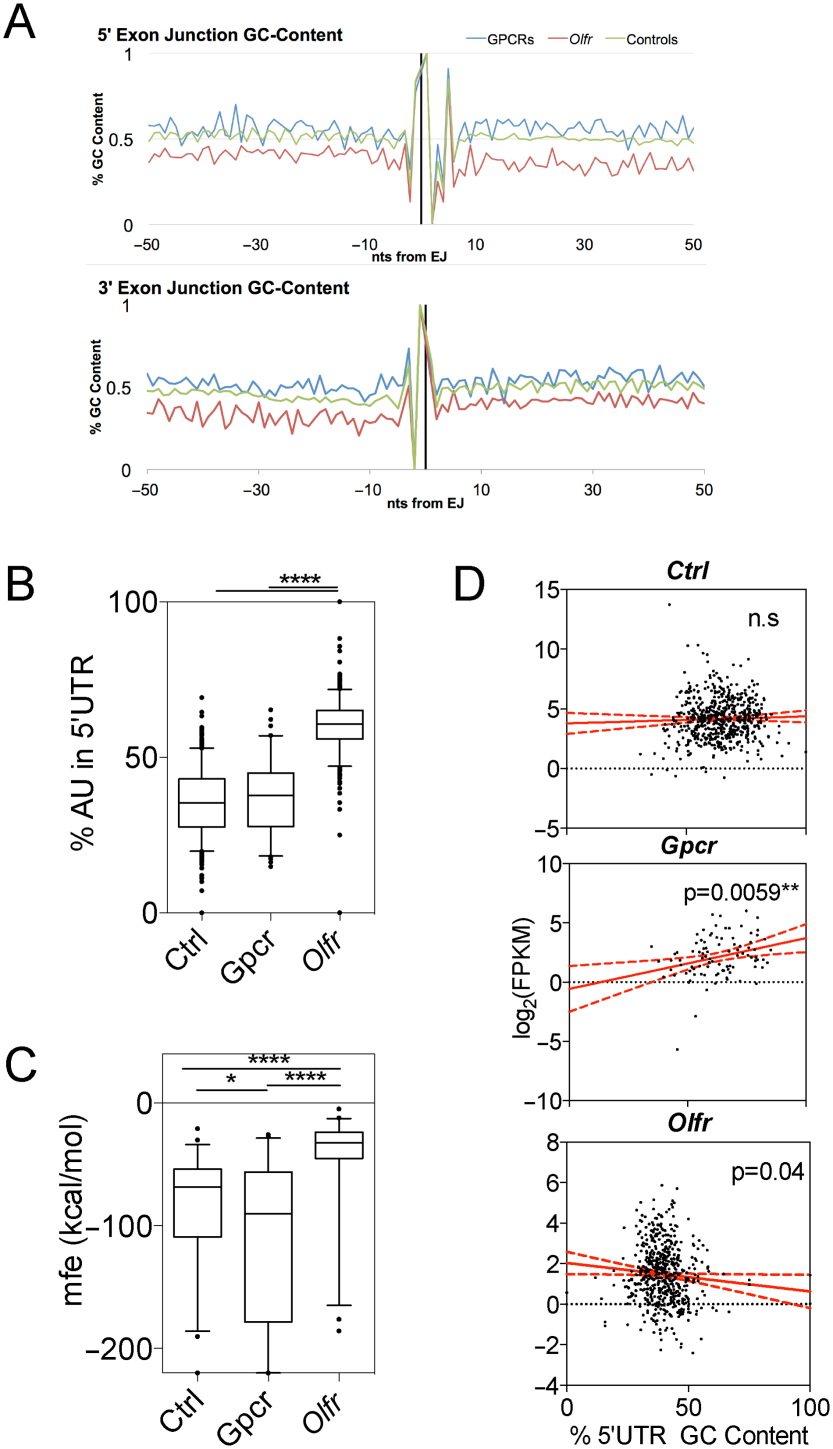
*Olfr* mRNAs are AU-rich. (**A**) GC-content along exon-intron junctions (top) and intron-exon junctions (bottom). (**B**) Boxplot of% of AU-content in the 5′ UTR. (**C**) Boxplot of minimal free energy (mfe) in the 5′ UTR. (**D**) Scatterplot and linear regression analysis of expression level versus 5′ UTR% GC-content. * *P* < 0.05; ** *P* < 0.01; **** *P* < 0.0001; n.s., not statistically significant.

Previous studies have demonstrated that the average AU-content of mammalian mRNAs is ∼45% (18). In contrast, we found that most *Olfr* mRNAs have an AU-content of greater than 50%. This characteristic was evident throughout the length of *Olfr* mRNAs, including both UTRs and the CDS, and even the splice junction region (Figure 4A–B, Supplementary Figure S2A–C). Particularly striking was the *Olfr* 5′ UTR region, which had ∼60% average AU-content versus the ∼40% average AU-content of the 5′ UTR region in the *Ctrl* and *Gpcr* mRNAs (Figure 4B, *P* < 0.0001, Supplementary Table S5). Of note, both class-I and –II *Olfr* mRNAs tended to be AU-rich (Supplementary Figure S2D–F), suggesting that this quality was independently selected for during the generation of both classes of *Olfr* genes.

The discovery that *Olfr* mRNAs have a high AU-content raised the possibility that this feature is important for *Olfr* post-transcriptional regulation. One possibility is that this reduces secondary structure that would otherwise reduce translation efficiency or lead to steric hindrance of interaction with RNA-binding proteins (19). To assess whether *Olfr* mRNAs have reduced secondary structure relative to control mRNAs, we utilized the RNAFold algorithm (37), which calculates minimum free energy (*mfe*), a measure of the difficulty to linearize a given sequence. Since 5′ UTR length contributes to *mfe* calculations, we selected 50 5′ UTRs from each mRNA group that have similar 5′ UTR length. This analysis revealed that *Olfr* mRNAs have a strikingly higher average *mfe* compared to *Gpcr* and *Ctrl* transcripts (Figure 4C).

mRNAs with low GC-content and secondary structure are thought to be more efficiently translated than those with a high GC-content (19). This is supported by genome-wide studies observing a significant correlation between 5′ UTR secondary structure folding free energy and protein abundance (62). While the molecular mechanism for this is not known, a likely possibility is that less structured 5′ UTRs permit more efficient scanning of the 40S ribosomal subunit as it traverses from the 5′ cap to the start AUG codon of the main ORF (63). This raises the possibility that *Olfr* genes have been selected to have low GC-content over evolutionary time so that they can translate high levels of *Olfr* proteins. Because translation can protect mRNAs from decay (64), this also predicts that *Olfr* mRNAs harboring 5′ UTRs with low GC-content would be more stable than those without. In agreement with this prediction, we found a negative correlation between *Olfr* expression level and the 5′ UTR GC-content of *Olfr* mRNAs (Figure 4D). In contrast, we observed a statistically significant *positive* correlation between the GC-content of *Gpcr* mRNAs and their expression. While we do not know the underlying mechanism for this, one possibility is higher GC-content alters the spectrum of ribonucleoprotein complexes that form on the 5′ UTR of *Gpcr* mRNAs in a manner that favors the stability of these mRNAs. No correlation was observed for the *Ctrl* mRNAs, possibly because they represent a broad group of genes with confounding variables. While there are several explanations for these findings, we suggest that the most parsimonious interpretation is that *Olfr* mRNAs evolved to have unstructured 5′ UTRs as a means to increase their translation efficiency, which in turn increased the stability of their mRNAs.

### *Olfr* mRNAs tend to be enriched for ARE motifs

Our finding that *Olfr* mRNAs tend to be AU-rich (Figure 4 and Supplementary Figure S2) raised the possibility that they are enriched for AU-rich binding elements (AREs). AREs are typically permutations of the core sequence AUUUA (or, more rarely, short stretches of U) that serve as *cis* elements to promote the degradation of mRNAs through a deadenylation-dependent mechanism called ARE-mediated RNA decay (AMD) (65,66).

**Figure 5.**
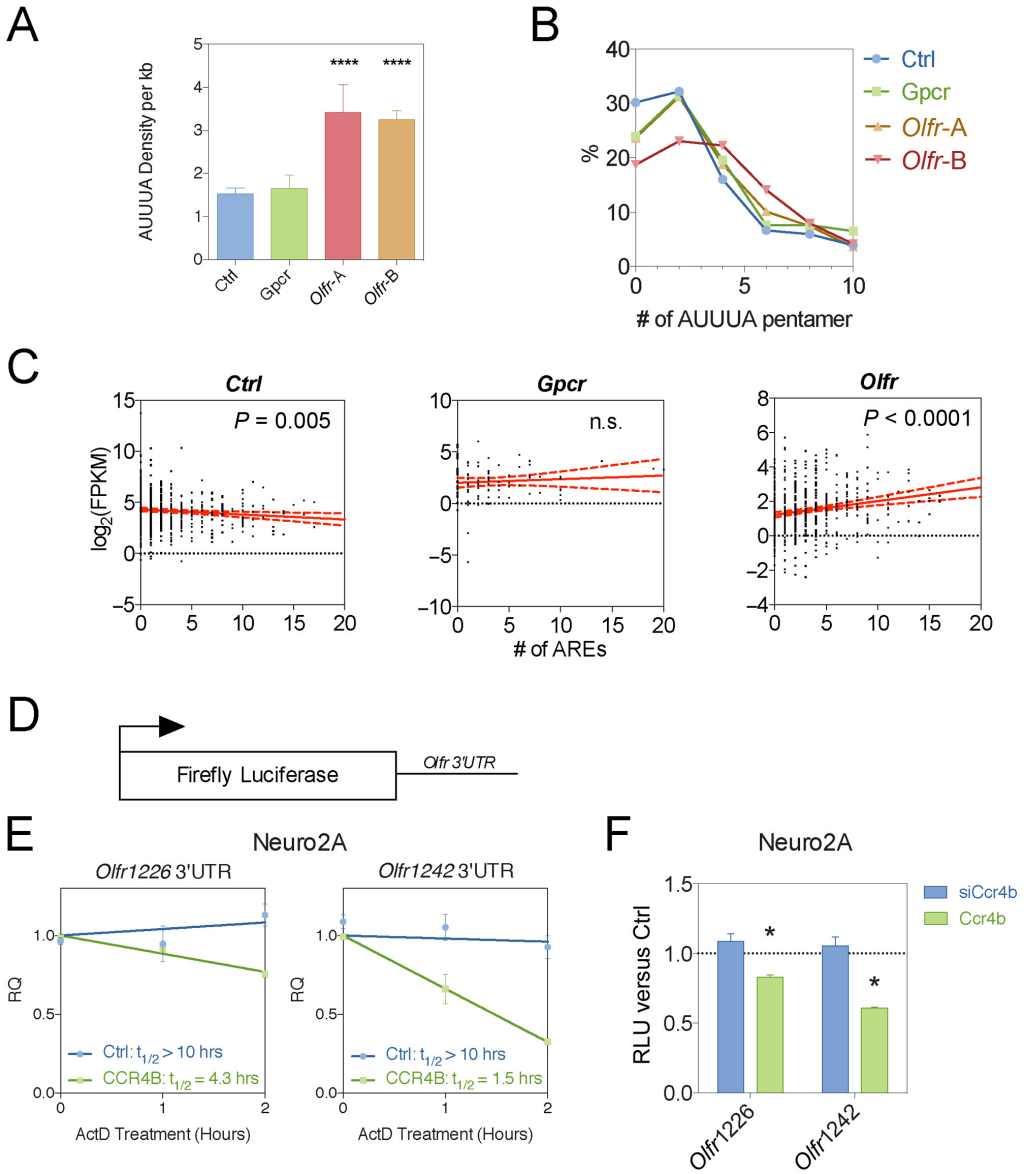
*Olfr* mRNAs are ARE-rich. (**A**) ARE pentamer density measured *in silico*. (**B**) Density distribution plot of the number of AUUUA ARE pentamers, assessed as in panel (**A**). (**C**) Scatterplots and linear regression analysis of expression level versus AUUUA pentamer density, assessed as in panel A. (**D**) Schematic of luciferase and renilla reporters used. (**E,F**) Two *Olfr* 3′ UTRs influence mRNA steady-state level and mRNA half-life in response to CCR4B depletion (using a siRNA targeting CCR4B [siCcr4b]) and CCR4B overexpression (using a CCR4B expression vector). Panel **E** is measurement of relative mRNA levels (RQ) and panel (**F**) is measurement of relative luciferase values (RLU). *P < 0.05; n.s. not significant.

AREs have been computationally defined in mammalian transcriptomes and deposited in several databases, including ARED and AREsite (32,67). However, these databases do not contain most *Olfr* mRNAs, including those annotated recently (28). Our annotation of *Olfr* genes expressed in the mouse OE together with another data set provided an opportunity to fill this gap. We analyzed the average ARE density in *Olfr* mRNAs and found it is twice as high as in the *Gpcr* and *Ctrl* mRNA groups (Figure 5A, Supplementary Table S6). We found this to also be the case when we analyzed the recently defined *Olfr* mRNA data set from Ibarra-Soria *et al*. (28) (‘*Olfr*-B’ in Figure 5A). Given that *Olfr* mRNAs have much shorter average 3′ UTR length than these other groups (see below), this is consistent with the possibility that there has been strong selection pressure for enrichment of AREs to maintain regulation by ARE-binding proteins. The net result is that AREs are as prevalent in *Olfr* mRNAs as in the other gene groups (Figure 5B).

To assess the possible impact of AREs on *Olfr* expression, we asked whether there is an association between ARE density and expression level. We found a strong positive correlation between AUUUA pentamer density and expression level, as assessed by RNA-seq reads (Figure 5C). Linear regression analysis demonstrated that this was highly statistically significant (*P* < 0.0001). In contrast, there was a negative correlation between ARE density and expression for the *Ctrl* group and no significant correlation for the *Gpcr* group (Figure 5C). To extend this analysis, we evaluated the density of another functional ARE motif: the WWAUUUAWW nonamer (32). As with AUUUA pentamer density, WWAUUUAWW density was higher in *Olfr* mRNAs than in the other groups (Supplementary Figure S3A). The frequency of these nonomers per transcript positively correlated with *Olfr* mRNA expression level (Supplementary Figure S3B).

Our finding that *Olfr* 3′ UTRs are both enriched in AREs and expressed in a pattern that positively correlates with ARE density raises the possibility that *Olfr* mRNAs are a new class of transcripts that are positively regulated by ARE-binding proteins. While AREs are normally associated with decay, they can also promote mRNA stabilization. Some ARE-binding proteins serve as mRNAs stabilizers, including members of the embryonic lethal abnormal vision (ELAV) family member (68). At least one member of this family, HuC, is expressed in the developing OE and other neuronal tissues (69). Another family member, HuD, is neuron-specific in its expression and is responsible for regulating *Gap43* mRNA, which encodes a neuron-specific protein critical for neurogenesis (70,71). This raised the possibility that AREs promote the expression of transcripts in a neuronal setting.

### *Olfr* 3′ UTRs confer stability

We examined whether the unique 3′ UTR characteristics of *Olfr* mRNAs confer stable expression in neural cells. To test this possibility, we cloned the 3′ UTRs of two *Olfr* genes—*Olfr1226* and *Olfr1242*—downstream of the firefly luciferase ORF in the pMIR-REPORT reporter vector (Figure 5D). We transfected these constructs into a neuronal cell line, Neuro2A, together with an endogenous control containing a Renilla plasmid reporter and measured the stability of the encoded transcripts. We found that both the *Olfr1226* and *Olfr1242* 3′ UTRs confer an extremely long RNA half-life in Neuro2A cells, as measured by treatment with the transcriptional inhibitor, Actinomycin D (>10 h; Figure 5E). This suggests that these 3′ UTRs confer stability to *Olfr* mRNA in neuronal cells.

A key rate-limiting step of mRNA decay is deadenylation (65). To examine whether *Olfr* 3′ UTRs confer deadenylation-dependent mRNA downregulation, we measured the effect of depleting or overexpressing the core deadenylase, CCR4B, on reporter mRNAs harboring the two *Olfr* 3′ UTRs described above. Forced overexpression of CCR4B (Supplementary Figure S3C) downregulated *Olfr* 3′ UTR-dependent reporter expression and destabilized the reporter mRNA transcript in Neuro2A cells (Figure 5E–F), suggesting that, indeed, *Olfr* 3′ UTRs are sensitive to deadenylation-dependent decay. In contrast, siRNA-mediated knockdown of CCR4B (Supplementary Figure S3C) did not significantly affect *Olfr* 3′ UTR-dependent reporter expression (Figure 5F), consistent with our finding that *Olfr* 3′ UTRs are normally stable in neural cells (Figure 5E).

To assess the specificity of this response, we tested whether *Olfr* 3′UTR responds to deadenylation-dependent decay in non-neuronal cells. We used P19 embryonal carcinoma cells for this experiment, which are stem-like cells that transfect efficiently (72). As we observed in Neuro2A cells, overexpression of CCR4B from an expression vector in P19 cells (Supplementary Figure S3D) elicited reduced reporter expression from the two *Olfr* 3′ UTR reporters (Supplementary Figure S3E). In striking contrast to the results obtained with Neuro2A cells, knocking down CCR4B in P19 cells (Supplementary Figure S3D) increased the expression the two *Olfr* 3′UTR reporters and stabilized its mRNA half-life (Supplementary Figure S3E and F). The difference in *Olfr* 3′ UTR sensitivity to CCR4B depletion in neuronal versus non-neuronal cells suggests that the *Olfr* 3′ UTRs have evolved to permit stable mRNA expression specifically in neural cells.

### *Olfr* mRNAs are uORF-rich

Another well-studied deadenylation-dependent RNA degradation mechanism is Nonsense-mediated RNA decay (NMD). This pathway that was originally identified by virtue of its ability to degrade aberrant mRNAs containing premature stop codons as a result of mutations or biosynthetic (e.g. RNA splicing) errors (73,74). More recently, it was discovered that NMD also targets normal mRNAs that have an in-frame stop codon in a context that is recognized as premature (73,75,76). Increasing evidence indicates that the ability of NMD to degrade a subset of normal mRNAs acts as a biological switch to control biological events, including developmental pathways (38,75–80). Thus, we investigated whether *Olfr* mRNAs might be targeted for decay by NMD.

The best-characterized context that triggers NMD is the presence of an exon-exon junction (intron) at least 55 nt downstream of the stop codon defining the end of the main ORF (81). Most *Olfr* mRNAs do ‘not’ have this architecture (Supplementary Figure S4). Nevertheless, we identified a small number of *Olfr* mRNAs (15 of 805) defined by RNA-seq analysis that do possess an ‘NMD-inducing’ architecture; i.e. an exon-exon junction >55 nt downstream of the stop codon (Supplementary Figure S4). Since virtually all mRNAs with this feature are targeted for decay by NMD (73,74), it is likely that this small subset of *Olfr* transcripts is subject to rapid decay by NMD, perhaps to provide developmental regulation.

Another NMD-inducing feature is the presence of a long 3′ UTR (82,83). While no specific 3′ UTR length has been shown to trigger NMD, our finding that *Olfr* mRNAs tend to have considerably shorter 3′ UTRs than do control mRNAs (Figure 2A) suggests that it is unlikely that *Olfr* transcripts are generally degraded by this mechanism.

**Figure 6.**
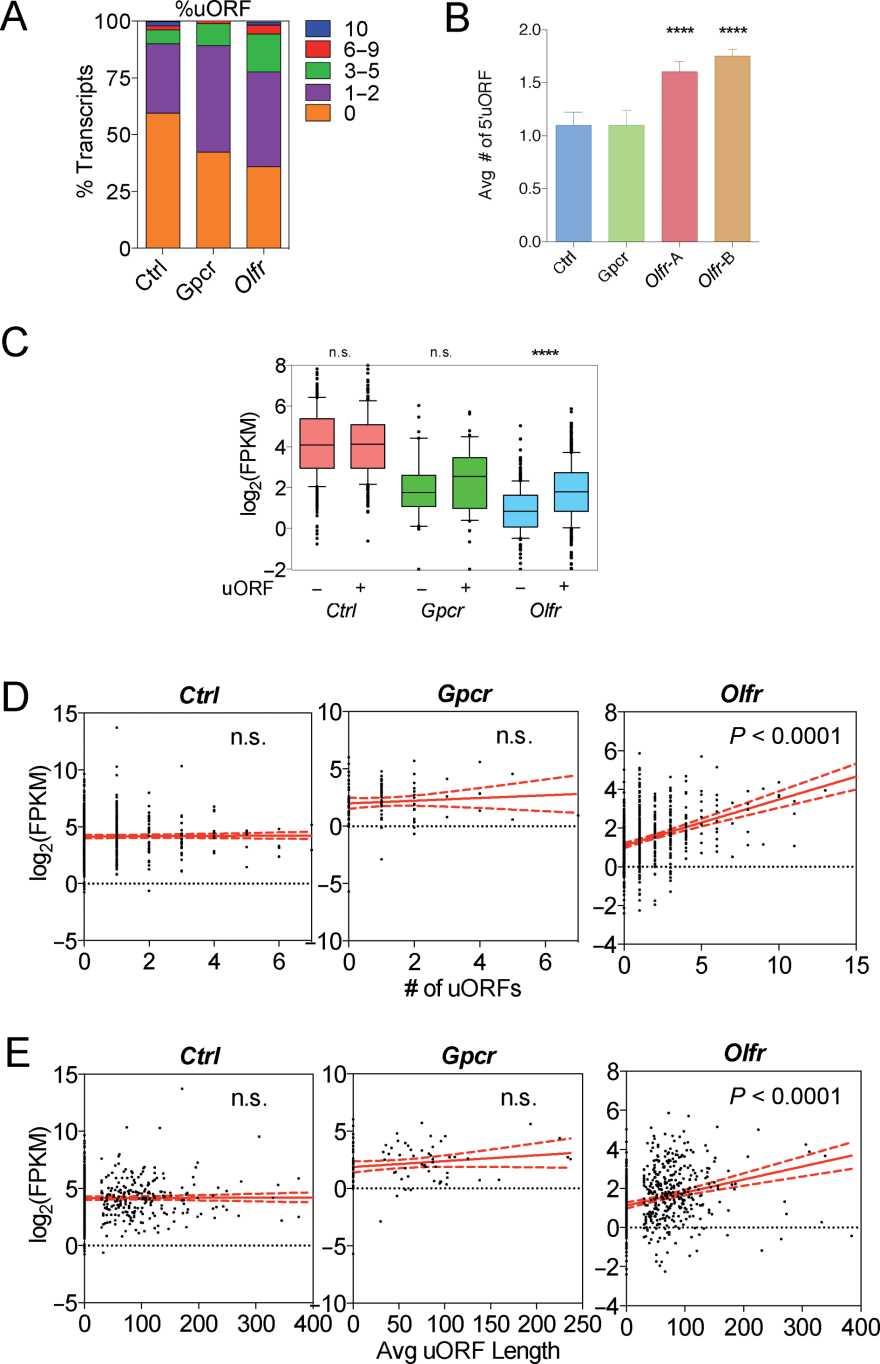
*Olfr* mRNAs are uORF-rich. (**A**) The percentage of transcripts from the three groups with the indicated number of uORFs. (**B**) The average number of uORFs. **(C)** Box plot comparing the expression level of transcripts harboring or lacking uORFs from the three groups. (**D**) Scatterplot and linear regression analysis of expression level versus uORFs density. (**E**) Scatterplot and linear regression analysis of expression level versus average uORF length. **** *P* < 0.0001; n.s., not statistically significant.

Additionally, it has been shown that upstream open reading frames (uORFs) can trigger NMD, perhaps by virtue of the ability of the stop codon in the uORF to trigger premature translation termination and thereby recruit mRNA decay enzymes (19,74). We found that majority of the 805 *Olfr* mRNAs expressed in the OE (67%) have uORFs (Figure 6A, Supplementary Table S7). This is a significantly higher proportion than in the *Gpcr* and *Ctrl* groups. Only 38% of the *Ctrl* mRNA group have uORFs, a value consistent with the average value recently reported for all human mRNAs (84). In addition, *Olfr* mRNAs from both our data set and Ibarra-Soria *et al*. have a significantly higher average number of uORFs per transcript than *Ctrl* and *Gpcr* group (Figure 6B) despite having a similar average 5′ UTR length as the *Ctrl* and *Gpcr* groups (Figure 2C).

Even though uORFs *can* trigger NMD, few uORF-containing mRNAs are likely to be subject to NMD regulation (85). This follows from the fact that while ∼40% of human and rodent mRNAs have uORFs (84,86,87) (Figure 6A), at most 5% of mammalian mRNAs are targeted for decay by NMD, based on microarray and RNA-seq analyses of cells depleted of NMD factors (85). Indeed, several mRNAs have been defined that escape uORF-induced NMD (88,89). This predicts that a majority of uORFs found in *Olfr* mRNAs are not *bona fide* targets of NMD. Consistent with this prediction, we found that *Olfr* mRNA harboring uORFs were statistically more highly expressed, when analyzed as a group, than those without (Figure 6C). We also observed a statistically significant positive correlation between the number of uORFs per transcript and *Olfr* mRNA level (Figure 6D). Finally, we also observed a positive correlation between average uORF length and *Olfr* mRNA level (Figure 6E). As evidence for specificity, we did not observe a correlation between any of these parameters and the expression of the *Gpcr* or *Ctrl* gene groups (Figure 6C–E). Together, these results suggest that uORFs have an overall positive effect on *Olfr* mRNA expression. It will be interesting in the future to determine the underlying mechanism. One means by which uORF-containing mRNAs may escape uORF-induced NMD is by allowing ribosomes that terminate translation in uORFs to reinitiate downstream. In *S. cerevisiae*, the 5′ UTR environment surrounding uORFs has been shown to influence this. For example, it has been shown that the AU-rich region downstream of the uORF in *gcn4* mRNA allows for efficient ribosome reinitiation, thereby avoiding triggering NMD (90). Given that we found that *Olfr* 5′ UTRs tend to be AU-rich (Figure 4), this may provide a general mechanism by which they escape uORF-induced NMD. Given that translation is generally regarded as providing a protective role from mRNA degradation (64), one explanation is that the AU-rich environment of *Olfr* uORFs promotes stabilization of *Olfr* mRNAs through their ability to promote re-initiation of translation. We note, however, that our results do not rule out that a subset of *Olfr* mRNAs are targeted for decay through their uORFs by NMD.

## SUMMARY

A comprehensive landscape of mRNA features present in *Olfr* mRNAs revealed several unusual mRNA features in both the 5′ and 3′ UTR of *Olfr* mRNAs that we posit were selected over evolutionary time to direct the post-transcriptional regulation of *Olfr* genes. We provide evidence that *Olfr* mRNAs are extremely stable, which we posit results from one or more of these post-transcriptional features. Mechanisms potentially impacted by the unique features in Olfr mRNAs include ARE-mediated RNA decay, miRNA-mediated regulation and NMD.

## SUPPLEMENTARY DATA

Supplementary Data are available at NAR Online.

SUPPLEMENTARY DATA
