# Supplementary material for: Identification of novel post-transcriptional features in olfactory receptor family mRNAs
Source: Nucleic Acids Res. 2015 Apr 23;43(19):9314–26. doi: 10.1093/nar/gkv324 (PMC4627058; doi:10.1093/nar/gkv324)
Supplement: SUPPLEMENTARY DATA [file supp_43_19_9314__index.html]

Identification of novel post-transcriptional features in olfactory receptor family mRNAs — SUPPLEMENTARY DATA 

# Identification of novel post-transcriptional features in olfactory receptor family mRNAs

## SUPPLEMENTARY DATA

- SUPPLEMENTARY DATA
- SUPPLEMENTARY DATA
