# Supplementary figures and images for: Identification of novel post-transcriptional features in olfactory receptor family mRNAs
Source: Nucleic Acids Res. 2015 Apr 23;43(19):9314–26. doi: 10.1093/nar/gkv324 (PMC4627058; doi:10.1093/nar/gkv324)

Figure S1

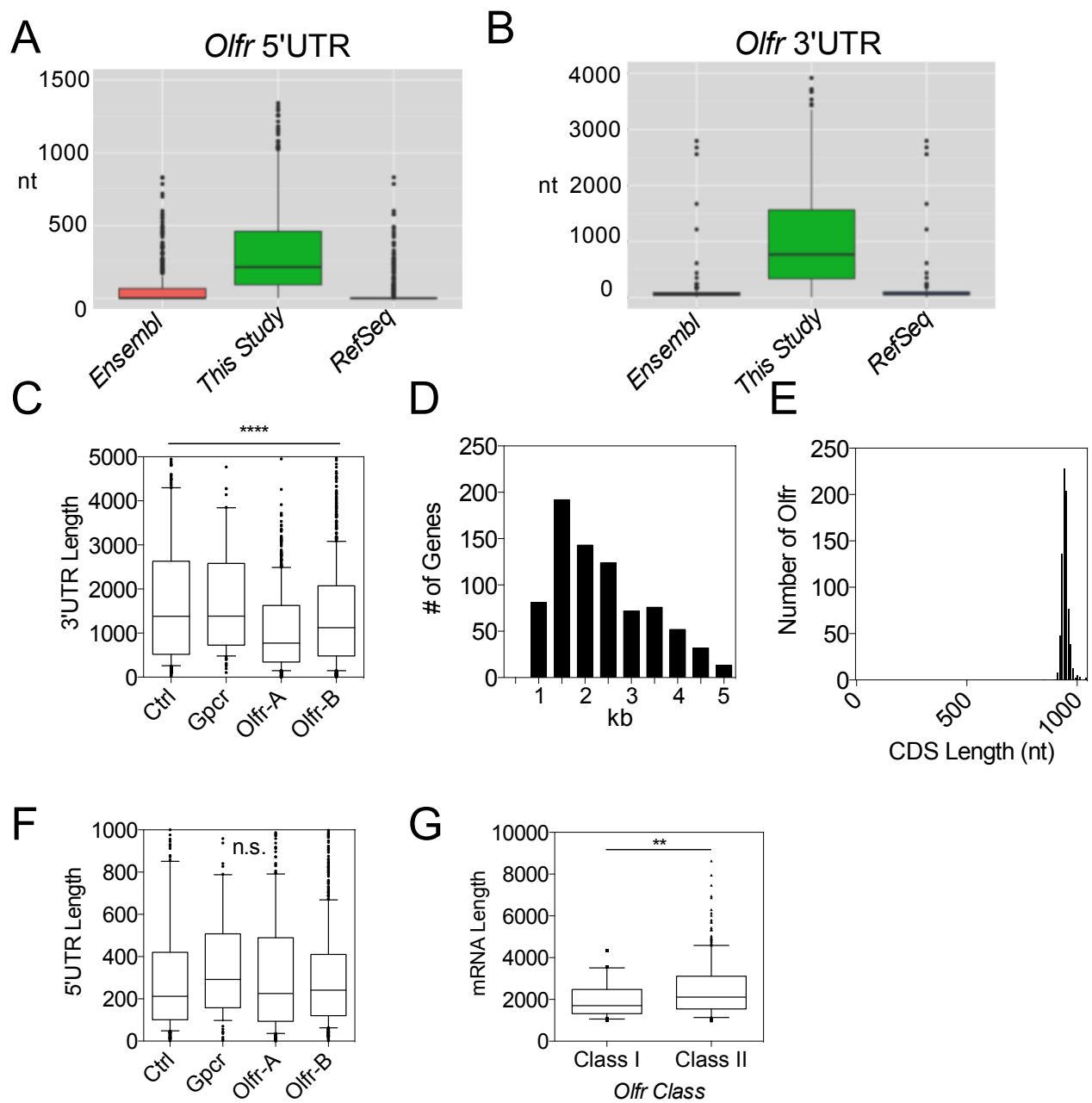

Figure S2

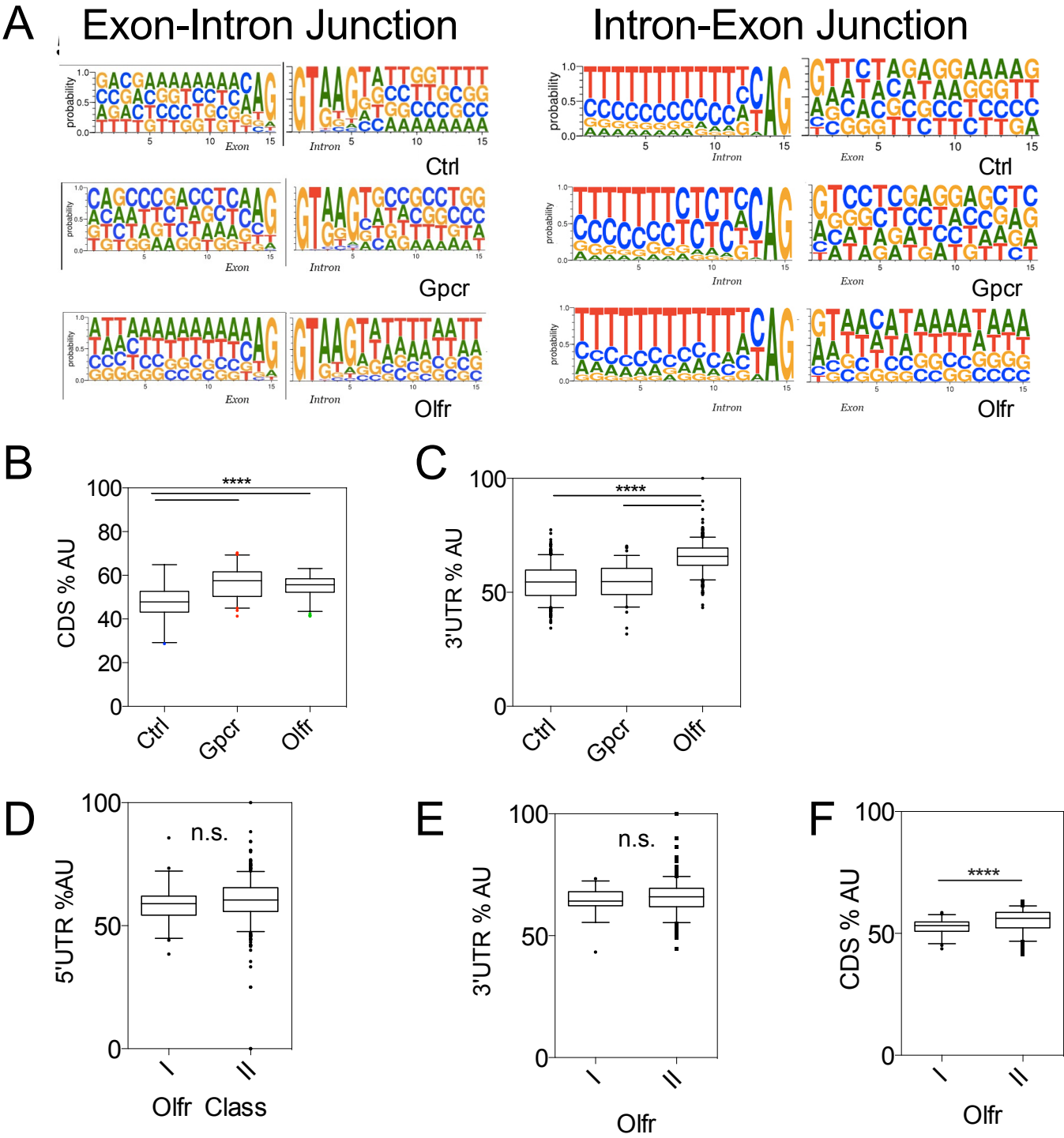

Figure S3

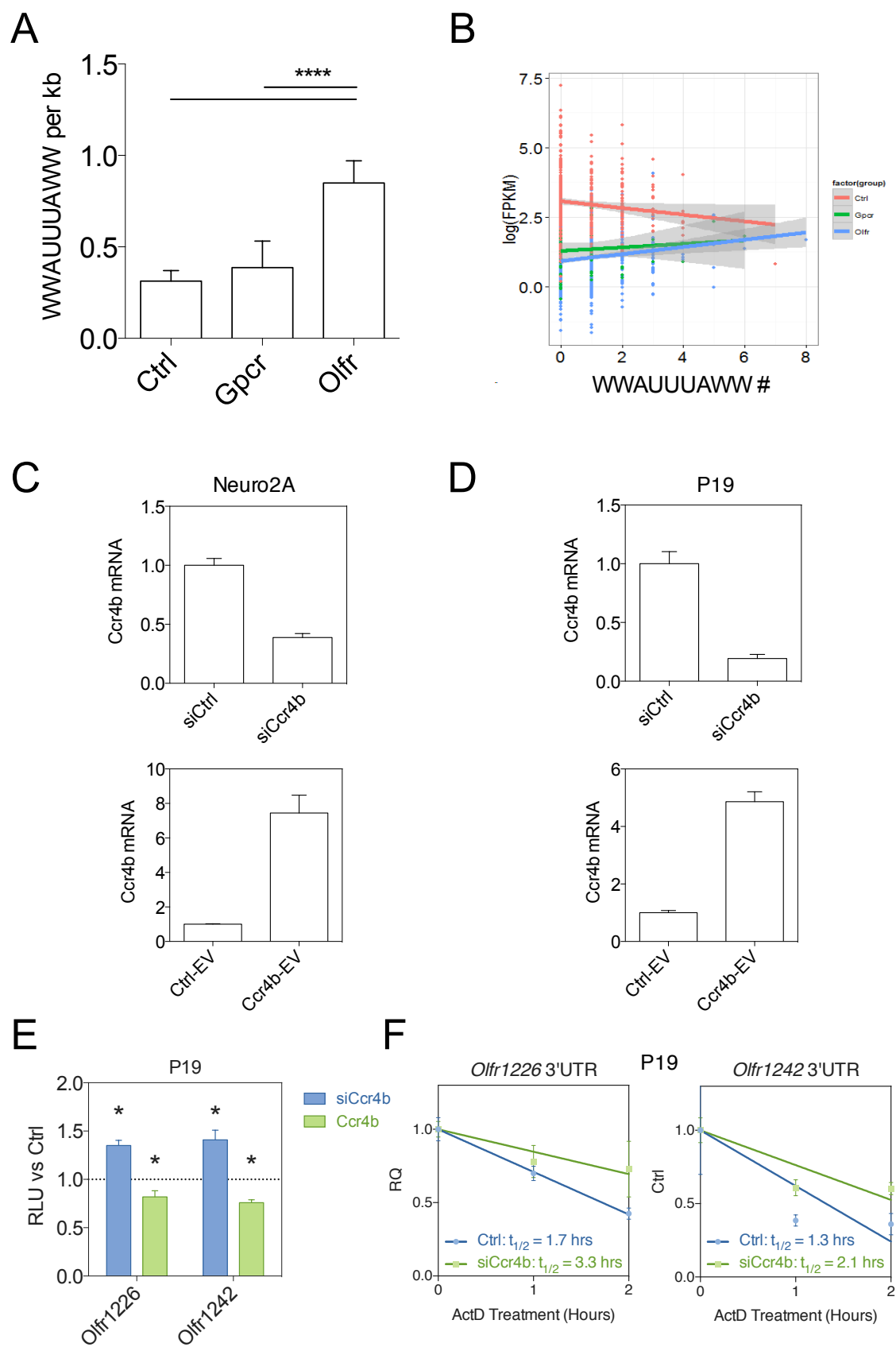

Figure S4

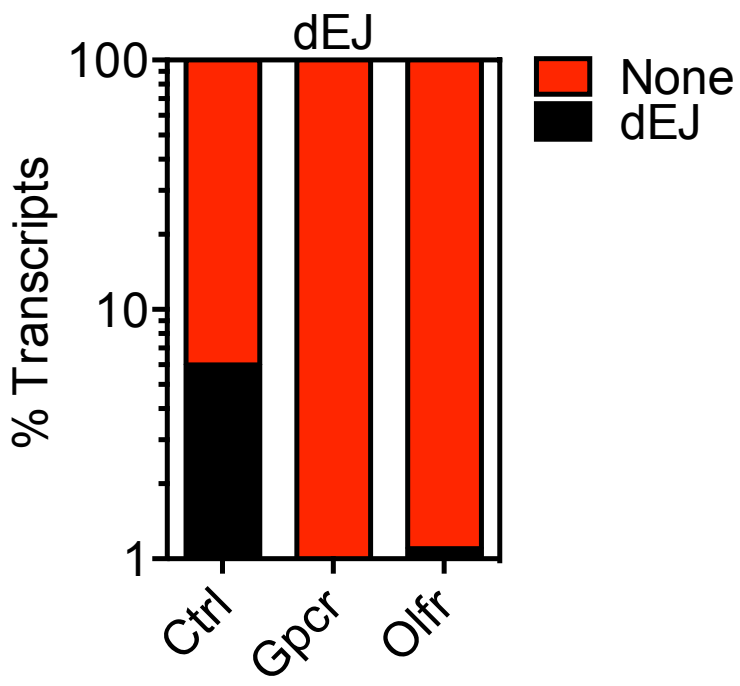

Supplement: SUPPLEMENTARY DATA [file supp_gkv324_Supplementary_Figures.pdf]
