# Supplementary material for: Identification of novel post-transcriptional features in olfactory receptor family mRNAs
Source: Nucleic Acids Res. 2015 Apr 23;43(19):9314–26. doi: 10.1093/nar/gkv324 (PMC4627058; doi:10.1093/nar/gkv324)
Supplement: SUPPLEMENTARY DATA [file supp_gkv324_Supplementary_Figure_Legends.docx]

**Identification of Novel Post-Transcriptional Features in**

**Olfactory Receptor Family mRNAs**

Eleen Y. Shum^1^, Josh L. Espinoza^1^, Madhuvanthi Ramaiah^1^, Miles F. Wilkinson^1,2,^

^1^Department of Reproductive Medicine, School of Medicine, University of California, San Diego, La Jolla, CA 92093-0695, USA

^2^Institute of Genomic Medicine, University of California, San Diego, La Jolla, CA 92093, USA

**SUPPLEMENTARY FIGURE LEGENDS**

**Supplementary Figure 1.** *Olfr* mRNA characterization. **(A**, **B)** *Olfr* UTR lengths defined by the RNA-seq analysis in our study versus those currently annotated in Ensembl and RefSeq (versions NCBIM37 and mm10, respectively). **(C, F)** Boxplot displaying lengths of **(C)** 3’ UTR and **(F)** 5’ UTR in gene groups indicated. **(D)** Distribution of *Olfr* mRNAs lengths defined by our RNA-seq analysis. **(E)** Distribution of coding sequence (CDS) lengths in *Olfr* mRNAs defined by our RNA-seq analysis. **(G)** Boxplot displaying lengths of class-I and -II *Olfr* full-length mRNAs defined by our RNA-seq analysis. ** p <0.01. **** p<0.0001. n.s. not significant.

**Supplementary Figure 2.** *Olfr* transcripts are AU-rich. **(A)** Nucleotide frequency around exon-intron (left) and intron-exon junctions (right) in each gene group, as determined using Weblogo. **(B**-**F)** Boxplots of % AU content in the indicated regions of the RNAs expressed from the gene groups indicated.

**Supplementary Figure 3.** *Olfr* transcripts are ARE rich. (**A**) Density of the ARE motif, WWAUUUAWW, in the 3’ UTRs of the 3 gene groups shown. (**B**) Scatterplot and linear regression analysis of the expression level versus the frequency of the WWAUUUAWW element. Only the *Olfr* gene group has a statistically significant positive correlation. **(C, D)** *Ccr4b* mRNA level in the different treatment groups. (**E,F**) Two *Olfr* 3’ UTRs influence mRNA steady-state level and mRNA half-life in response to CCR4B depletion (using a siRNA targeting CCR4B [siCcr4b]) and CCR4B overexpression (using a CCR4B expression vector). Panel **E** is measurement of relative luciferase values (RLU) and panel **F** is measurement of relative mRNA levels (RQ). *p<0.05; ****p <0.0001.

**Supplementary Figure 4.** Percentage of transcripts in each gene group that have 1 or more exon-exon junction >55 nt downstream of the stop codon ending the main open reading frame, a context that triggers NMD. dEJ (downstream exon-exon junction) refers to transcripts with this NMD-inducing feature.
